# Integrated stress response is involved in the 24(*S*)-hydroxycholesterol-induced unconventional cell death mechanism

**DOI:** 10.1038/s41420-022-01197-w

**Published:** 2022-10-04

**Authors:** Yasuomi Urano, Shoya Osaki, Ren Chiba, Noriko Noguchi

**Affiliations:** Department of Medical Life Systems, Faculty of Life and Medical Sciences, Doshisha University, Kyoto, 610-0394 Japan

**Keywords:** Sterols, Apoptosis

## Abstract

Perturbation of proteostasis triggers the adaptive responses that contribute to the homeostatic pro-survival response, whereas disruption of proteostasis can ultimately lead to cell death. Brain-specific oxysterol—i.e., 24(*S*)-hydroxycholesterol (24S-OHC)—has been shown to cause cytotoxicity when esterified by acyl-CoA:cholesterol acyltransferase 1 (ACAT1) in the endoplasmic reticulum (ER). Here, we show that the accumulation of 24S-OHC esters caused phosphorylation of eukaryotic translation initiator factor 2α (eIF2α), dissociation of polysomes, and formation of stress granules (SG), resulting in robust downregulation of global protein *de novo* synthesis in human neuroblastoma SH-SY5Y cells. We also found that integrated stress response (ISR) activation through PERK and GCN2 activation induced by 24S-OHC treatment caused eIF2α phosphorylation. 24S-OHC-inducible SG formation and cell death were suppressed by inhibition of ISR. These results show that ACAT1-mediated 24S-OHC esterification induced ISR and formation of SG, which play crucial roles in 24S-OHC-inducible protein synthesis inhibition and unconventional cell death.

## Introduction

Cellular protein homeostasis, which is also known as proteostasis, is essential for living cells to maintain their normal cellular function [1]. Proteostasis is a delicate intracellular balance of cellular protein levels between the synthesis of de novo proteins and the appropriate and efficient clearance of damaged and misfolded proteins. The dysregulation of proteostasis often leads to cellular dysfunction and pathophysiological states. Cellular stress precipitated by accumulation of damaged or misfolded proteins or exposure to external stimuli evokes specific stress responses, such as the heat-shock response, the unfolded protein response (UPR), and the integrated stress response (ISR) [2]. Activation of such pathways causes a reduction in global protein synthesis as well as transcriptional activation for translation of specific factors to restore proteostasis. A failure to restore the proteostasis network can bring about prolonged stress and cause activation of cell-death signaling cascades [3, 4].

The UPR, which is activated by the accumulation of unfolded/misfolded proteins in the endoplasmic reticulum (ER), is mainly an adaptive response that encompasses ISR activation [5, 6]. The UPR signalings are composed of increase in ER chaperones, downregulation of protein synthesis, and misfolded polypeptide degradation via ER-associated degradation (ERAD). In mammalian cells, the UPR employs three main signaling pathways, these being activating transcription factor 6 (ATF6), inositol-requiring enzyme 1 (IRE1), and protein kinase RNA-like ER kinase (PERK). Of these, the PERK pathway is responsible for a part of the ISR signaling pathway [6]. Activated PERK (via autophosphorylation) phosphorylates eukaryotic translation initiator factor 2α (eIF2α) at serine 51, thereby inhibiting ability of eIF2 to deliver initiator methionyl-tRNA to ribosomes and causing general attenuation of 5′ Cap-dependent protein synthesis [7]. Moreover, phosphorylation of eIF2α enhances specific translation of ATF4, which induces expression not only of genes that encode pro-survival proteins but also genes that encode proapoptotic proteins. In addition to PERK, the ISR involves three other eIF2α kinases, these being general control nonderepressible 2 (GCN2), heme-regulated eIF2α kinase (HRI), and double-stranded RNA-dependent protein kinase (PKR). These are respectively activated by amino acid deprivation and UV light, heme deficiency, and viral infection [2, 6].

Upon phosphorylation of eIF2α, when translation is initiated in the absence of a ternary complex consisting of eIF2, GTP, and initiator methionyl-tRNA, a stalled 48S pre-initiation complex results [8, 9]. This pre-initiation complex and the mRNA transcripts associated therewith bind to T-cell-restricted intracellular antigen-1 (TIA1), Ras-GAP SH3 domain-binding protein 1 (G3BP1), and/or other such RNA-binding proteins (RBPs) to form untranslated messenger ribonucleoproteins (mRNPs). The resulting complexes further assemble into membraneless cytoplasmic organelles, called stress granules (SGs) through liquid–liquid phase separation (LLPS). Dynamic equilibrium is maintained between SGs and polysomes. Upon recovering from stress, mRNPs within SGs may be redirected to translation or may be targeted for autophagy [10]. Persistent or aberrant SG formation is implicated in disease pathology and cell death [10, 11].

24(*S*)-Hydroxycholesterol (24S-OHC) is an enzymatically formed oxysterol that is catalyzed by the brain-specific cholesterol 24-hydroxylase (CYP46A1). As 24S-OHC is able to effectively pass through the blood–brain barrier, it plays a vital role for regulating brain cholesterol homeostasis [12–16]. Consistent with the physiological functions of 24S-OHC within the brain, dysregulation of 24S-OHC metabolism contributes to the development of Alzheimer’s disease (AD), Parkinson’s disease, and other such neurodegenerative diseases, as well as of glioblastoma [17–25]. We have reported that 24S-OHC induces caspase-independent unconventional cell death in SH-SY5Y human neuroblastoma, rat primary cortical neurons, and HepG2 human hepatic cells [26–28]. Our recent work further demonstrated that acyl-CoA:cholesterol acyltransferase 1 (ACAT1) [29] causes 24S-OHC to be esterified with unsaturated long-chain fatty acids, resulting in a situation in which such esters of 24S-OHC accumulate in ER, which in turn leads to abnormal ER morphology which is accompanied by disruption of ER membrane integrity [30–32]. ER dysfunction induced by 24S-OHC was accompanied by activation of pro-death UPR signaling including regulated IRE1-dependent mRNA decay (RIDD) [33] but was not accompanied by the pro-survival adaptive response. 24S-OHC treatment also evoked robust suppression of global protein *de novo* synthesis. Although ER dysfunction contributes to the 24S-OHC-induced cell death [32], the specific role of eIF2α in cell death signaling and protein synthesis inhibition remains unclear.

In the present study, we found that 24S-OHC esterification triggered eIF2α phosphorylation and SG formation. We further demonstrated that 24S-OHC induced ISR activation through PERK and GCN2 activation, which downregulated global protein *de novo* synthesis. We further found that this 24S-OHC-inducible SG formation and cell death could be suppressed by inhibition of ISR. Our results show that the side-chain oxysterol 24S-OHC induces ISR and SG formation, which are implicated in 24S-OHC-induced protein synthesis inhibition and cell death.

## Results

### Accumulation of 24S-OHC esters activated PERK signaling pathway and suppressed global protein synthesis

**Fig. 1:**
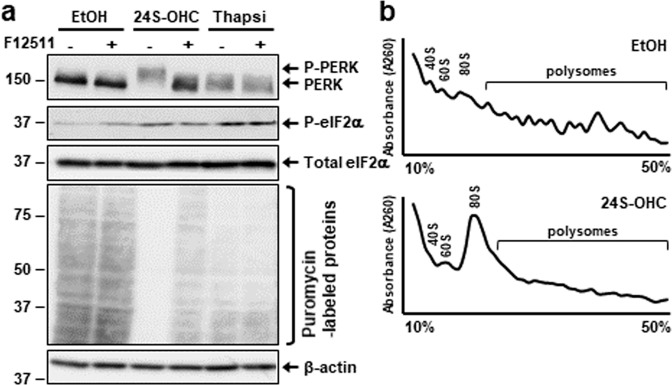
Accumulation of 24S-OHC esters activated PERK signaling pathway and downregulated global protein synthesis. **a** SH-SY5Y cells were pretreated with 5 μM F12511 for 15 min and then exposed for 3 h to 50 μM 24S-OHC or 1 μM thapsigargin (Thapsi). Cells were then incubated with 10 μg/ml puromycin for final 15 min. Whole-cell lysates were subjected to immunoblotting using appropriate antibodies, as indicated. **b** Cells were treated with or without 50 μM 24S-OHC for 3 h. Cell extracts were subjected to sucrose gradient polysome analyses. Monitoring of ribosomal distributions was carried out by measuring absorbance at 260 nm.

To investigate whether esterification by ACAT1 of 24S-OHC induces eIF2α activation in SH-SY5Y cells, we first used immunoblotting to investigate PERK and eIF2α phosphorylation. Phosphorylation of PERK, as observed in the upward shift, and the increase in phosphorylated eIF2α at Ser-51 occurred in cells treated with 24S-OHC for 3 h as compared with vehicle (EtOH) condition; moreover, these effects could be suppressed by cotreatment with the ACAT inhibitor F12511 (Fig. 1a). In contrast, thapsigargin, a potent ER stress inducer, triggered the phosphorylation of PERK and eIF2α; however, neither of them was suppressed by cotreatment with F12511. We then used SUnSET assay in which puromycin was incorporated in newly synthesized peptides followed by detection using an anti-puromycin antibody [34] to examine the effect of 24S-OHC on global protein *de novo* synthesis. Similar to our previous observation [32], we observed a dramatic decrease in puromycin-labeled proteins that was capable of being significantly suppressed by F12511 in cells treated with 24S-OHC, whereas the thapsigargin-induced moderate decrease that we observed in puromycin-labeled proteins was not inhibited by F12511.

Because phosphorylation of eIF2α causes the dissociation of polysomes and the accumulation of monosomes, leading to a decrease in translation initiation [35], we analyzed polysome profiles in 24S-OHC-treated cells (Fig. 1b). Results of analysis indicated that 24S-OHC treatment elevated the 80S monosome peak but reduced polysome fractions as compared with vehicle control. These results indicated that 24S-OHC esterification elicited not only PERK–eIF2α activation, but also suppression of global protein de novo synthesis.

### ACAT1-mediated 24S-OHC esterification triggered SG formation

**Fig. 2:**
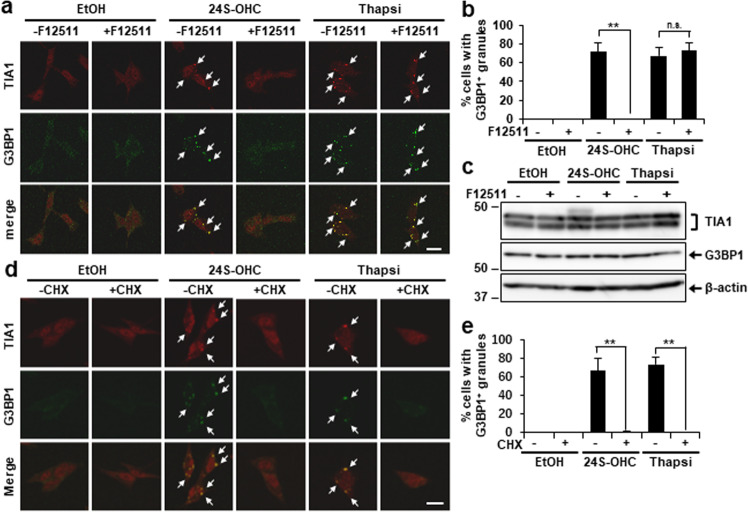
Accumulation of 24S-OHC esters induced SG formation. **a**, **b** Cells grown on cover slips were pretreated for 15 min with 5 μM F12511 and then exposed for 3 h to 50 μM 24S-OHC or 1 μM Thapsi. Cells were subjected to immunofluorescence staining for TIA1 and G3BP1. **a** Representative confocal images (white arrows indicate colocalization). Length of scale bar = 20 μm. **b** Percentage of cells containing G3BP1-positive SGs. **P < 0.01; n.s. not significant. **c** Cells were treated as in Fig. 2a. Immunoblot analysis of TIA1, G3BP1, and β-actin. **d**, **e** Cells grown on cover slips were treated with 50 μM 24S-OHC or 1 μM Thapsi for 3 h in presence or absence of 100 μM cycloheximide (CHX). Cells were subjected to immunofluorescence staining for TIA1 and G3BP1. **d** Representative confocal images. **e** Percentage of cells containing G3BP1-positive SGs.

As phosphorylation of eIF2α and reduction of de novo protein synthesis are tightly linked to formation of SGs [8, 9], we next examined the induction of SG formation in 24S-OHC-treated cells. We monitored SGs by immunocytochemical analysis using antibodies against TIA1 and G3BP1, which are representative markers of SGs. In a control experiment, we observed the colocalization of TIA1 and G3BP1 in cytoplasmic granules upon thapsigargin treatment compared with the vehicle condition, suggesting that thapsigargin induces the formation of SGs (Fig. 2a, b). Similar TIA1- and G3BP1-positive granules were observed in 24S-OHC-treated cells. Cotreatment with F12511 suppressed SG formation that would otherwise have been induced by 24S-OHC, but not by thapsigargin (Fig. 2a, b), without affecting the total amount of TIA1 (two isoforms of TIA1, TIA1a and TIA1b) and G3BP1 proteins (Fig. 2c). To confirm whether these cytoplasmic granules were indeed SGs, we evaluated the effects of cycloheximide (CHX), which blocks SG assembly by stabilizing polysomes; we found that cotreatment with CHX inhibited SG formation in cells that had been treated with 24S-OHC or thapsigargin (Fig. 2d, e). Together, the foregoing results suggest that treatment with 24S-OHC induces SG formation in an ACAT1-mediated 24S-OHC esterification-dependent manner.

We next wondered whether disease-associated proteins might also be recruited to SGs in response to 24S-OHC treatment. We therefore examined the localization of TAR DNA-binding protein 43 (TDP-43)—an amyotrophic lateral sclerosis (ALS)-associated protein—because TDP-43 is known to be recruited to SGs in response to neuronal injury [36]. As expected, endogenous TDP-43 was almost entirely localized to the nucleus under vehicle control conditions; but upon treatment with 24S-OHC, whereas TDP-43 continued to be mostly confined to the nucleus, a small amount of TDP-43 was observed in the cytoplasm, where it was found to colocalize with G3BP1-positive granules (Supplementary Fig. 1, white arrow). SGs with a lower TDP-43 signal were also observed (Supplementary Fig. 1, white arrowhead). Cotreatment with F12511 suppressed TDP-43 translocation in accordance with the disappearance of SGs, suggesting that TDP-43 recruitment to SGs is dependent on ACAT1-mediated 24S-OHC esterification.

### SG formation occurred concurrently with PERK phosphorylation and protein synthesis repression in cells treated with 24S-OHC

**Fig. 3:**
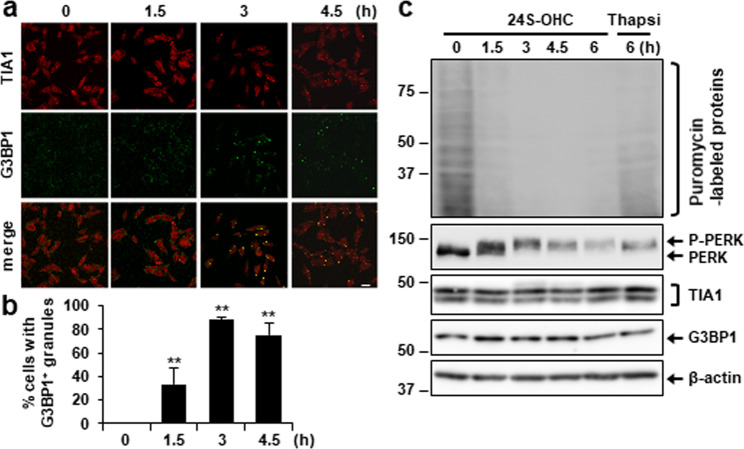
24S-OHC-induced SG formation occurred concurrently with 24S-OHC-induced protein synthesis repression. **a**, **b** Cells grown on cover slips were treated with 50 μM 24S-OHC for indicated periods. Cells were subjected to immunofluorescence staining for TIA1 and G3BP1. **a** Representative confocal images. **b** Percentage of cells containing G3BP1-positive SGs. **P < 0.01; compared with cells treated with the vehicle. **c** Cells were treated with 50 μM 24S-OHC for indicated periods or 3 μM Thapsi for 3 h. Cells were then incubated with 10 μg/ml puromycin for final 15 min. Whole-cell lysates were subjected to immunoblotting using appropriate antibodies, as indicated.

We next carried out a time-course study to ascertain the effect of 24S-OHC treatment on SG formation and de novo protein synthesis. An immunocytochemical analysis showed that SGs appeared at 1.5 h and became more prominent with time (Fig. 3a, b). In correlation with the progress of SG formation, puromycin-labeled proteins were significantly downregulated after the 1.5 h treatment, and became more marked with time (Fig. 3c). Phosphorylation of PERK also increased in time-dependent fashion. Throughout treatment, no significant change was observed in the level of TIA1 or of G3BP1. These results indicated that SG formation caused by 24S-OHC treatment occurred concurrently with activation of PERK and suppression of global protein *de novo* synthesis.

### Inhibition of the PERK pathway mitigated 24S-OHC-induced cell death

**Fig. 4:**
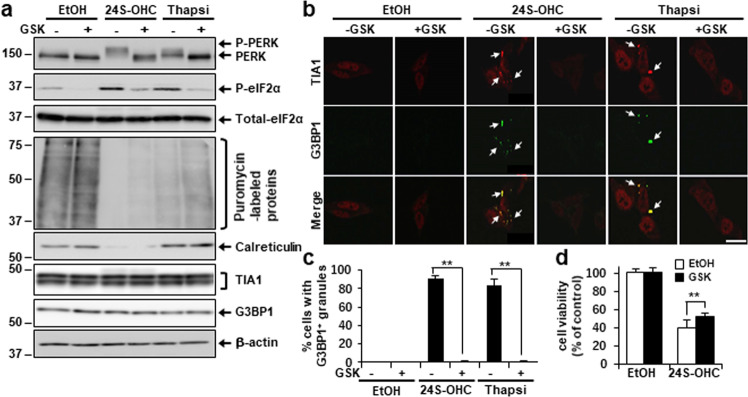
Inhibition of PERK axis suppressed 24S-OHC-induced SG formation and mitigated 24S-OHC-induced cell death. **a**–**c** Cells were treated with 50 μM 24S-OHC or 1 μM Thapsi for 3 h in presence or absence of 0.5 μM GSK2606414 (GSK). **a** Cells were then incubated with 10 μg/ml puromycin for final 15 min. Whole-cell lysates were subjected to immunoblotting using appropriate antibodies, as indicated. **b**, **c** Cells were subjected to immunofluorescence staining for TIA1 and G3BP1. **b** Representative confocal images. **c** Percentage of cells containing G3BP1-positive SGs. **P < 0.01. **d** Cells were treated with 30 μM 24S-OHC for 24 h in presence or absence of 0.5 μM GSK. Cell viability was measured by WST-8 assay.

We then sought to examine the possible role of PERK–eIF2a pathway activation in 24S-OHC-inducible SG formation and cell death. Whereas in our previous report [32] we had shown that the PERK inhibitor GSK2606414 at 10 μM did not suppress 24S-OHC-induced cell death; it later occurred to us that concentration of GSK2606414 tested there might have been too high, as treatment with 10 μM GSK2606414 alone yielded a slight reduction of cell viability. Therefore, we evaluated the effect of 0.5 μM GSK2606414, and found that GSK2606414 inhibited phosphorylation of PERK and reduced downstream phosphorylation of eIF2α in cells treated with 24S-OHC or thapsigargin (Fig. 4a). We observed a decrease in puromycin-labeled protein levels that was partially suppressed by treatment with GSK2606414, which suggested that PERK-regulated translation attenuation was implicated in inhibition by 24S-OHC of global protein synthesis. A decrease in calreticulin levels that we determined to have been caused by ER membrane disruption induced by 24S-OHC was unchanged by GSK2606414.

We further observed that GSK2606414 inhibited SG formation in cells treated with 24S-OHC or thapsigargin (Fig. 4b, c) without affecting TIA1 and G3BP1 expression (Fig. 4a). We also found that GSK2606414 could modestly but significantly inhibit cell death that otherwise would have resulted from treatment with 24S-OHC (Fig. 4d). Although induction of C/EBP-homologous protein (CHOP) is an important pro-death response in the PERK pathway, it should be noted that we previously demonstrated nonimplication of CHOP in the 24S-OHC-inducible cell death, based on the fact that we previously found CHOP expression to be only moderately induced and cell death to not be suppressed by knockdown of CHOP [32]. Taken together, the foregoing results indicate that activation of the PERK pathway which is accompanied by protein synthesis repression and SG formation is implicated in the 24S-OHC-induced cell death machinery.

### The GCN2–eIF2α axis was activated in 24S-OHC-treated cells

**Fig. 5:**
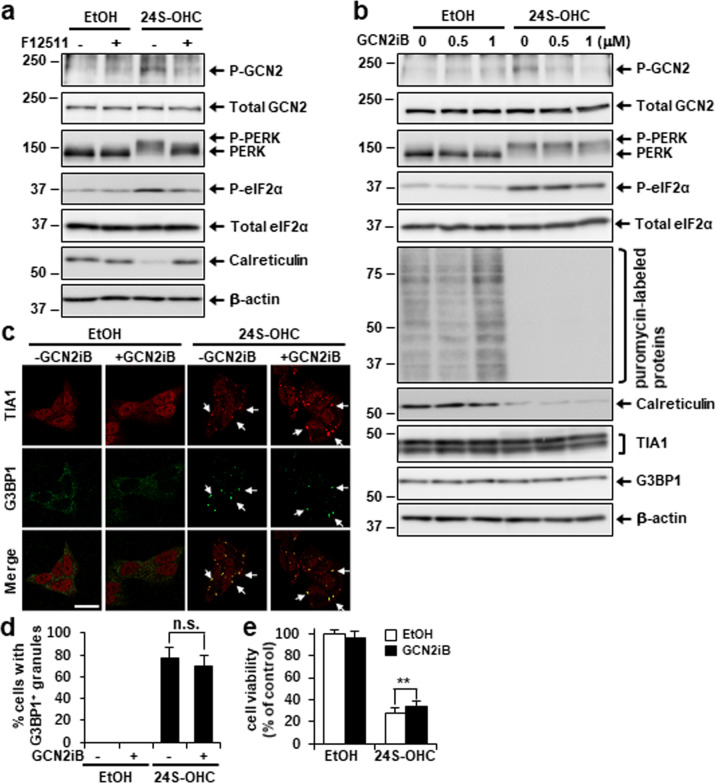
Inhibition of GCN2 axis alleviated 24S-OHC-induced cell death. **a** Cells were pretreated with 5 μM F12511 for 15 min and then exposed for 3 h to 50 μM 24S-OHC. Whole-cell lysates were subjected to immunoblotting using appropriate antibodies, as indicated. **b** Cells were treated with 50 μM 24S-OHC for 3 h in presence or absence of 0.5 or 1 μM GCN2iB. Cells were then incubated with 10 μg/ml puromycin for final 15 min. Whole-cell lysates were subjected to immunoblotting using appropriate antibodies, as indicated. **c**, **d** Cells grown on cover slips were treated with 50 μM 24S-OHC for 3 h in presence or absence of 1 μM GCN2iB. Cells were subjected to immunofluorescence staining for TIA1 and G3BP1. **c** Representative confocal images. **d** Percentage of cells containing G3BP1-positive SGs. n.s. not significant. **e** Cells were treated with 30 μM 24S-OHC for 24 h in presence or absence of 1 μM GCN2iB. Cell viability was measured by WST-8 assay. **P < 0.01.

Whereas 24S-OHC-inducible PERK phosphorylation was almost completely blocked by treatment with GSK2606414, a small amount of phosphorylated eIF2α was still observed (Fig. 4a, lane 4) in contrast to the robust reduction of phosphorylated eIF2α in GSK2606414-treated cells in the absence of 24S-OHC (Fig. 4a, lane 2). Therefore, we considered the possibility that another eIF2α kinase was also activated in response to 24S-OHC treatment. Because it has been reported that 25-hydroxycholesterol (25-OHC) treatment activates GCN2 in bone-marrow-derived macrophages [37], we evaluated GCN2 autophosphorylation upon 24S-OHC treatment, and found that GCN2 phosphorylation was observed in cells treated with 24S-OHC as compared with vehicle control, and that this could be suppressed by F12511 in a similar fashion to PERK and eIF2α phosphorylation (Fig. 5a). We further examined the effects of the GCN2 inhibitor, GCN2iB, and found that GCN2iB treatment inhibited GCN2 phosphorylation in a concentration-dependent manner, without affecting PERK phosphorylation (Fig. 5b). GCN2iB modestly inhibited eIF2α phosphorylation, but did not exhibit remarkable effect on the reduction in puromycin-labeled protein levels that was induced by 24S-OHC. We also observed that GCN2iB showed a mild but not significant inhibitory effect on SG formation in 24S-OHC-treated cells (Fig. 5c, d). In contrast, GCN2iB weakly but significantly suppressed cell death that would otherwise have been induced by 24S-OHC (Fig. 5e). Collectively, the foregoing results suggest that esterification of 24S-OHC caused GCN2 phosphorylation, which was partially involved in 24S-OHC-induced cell death.

### Inhibition of ISR suppressed 24S-OHC-inducible SG formation and cell death

**Fig. 6:**
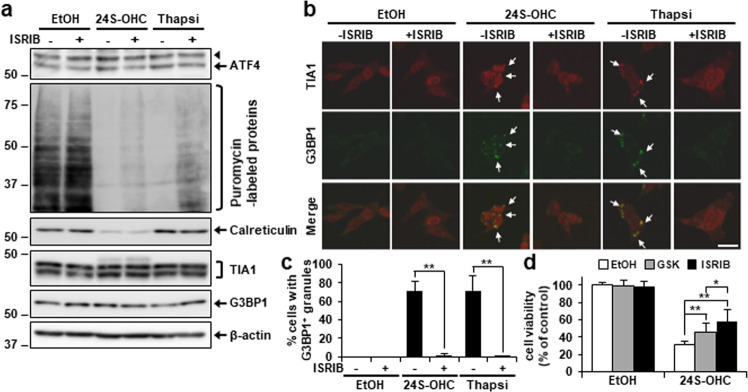
Inhibition of ISR suppressed 24S-OHC-inducible SG formation and cell death. **a**–**c** Cells were treated with 50 μM 24S-OHC or 1 μM Thapsi for 3 h in presence or absence of 200 nM ISRIB. Cells were then incubated with 10 μg/ml puromycin for final 15 min. Whole-cell lysates were subjected to immunoblotting using appropriate antibodies, as indicated. The arrowhead indicates a non-specific band. **b**, **c** Cells were subjected to immunofluorescence staining for TIA1 and G3BP1. **b** Representative confocal images. **c** Percentage of cells containing G3BP1-positive SGs. **P < 0.01. **d** Cells were treated with 30 μM 24S-OHC for 24 h in presence or absence of 0.5 μM GSK or 200 nM ISRIB. Cell viability was measured by WST-8 assay.

As both PERK and GCN2 were found to be activated in cells treated with 24S-OHC, we took this to indicate possible activation of the ISR. To examine the involvement of ISR signaling, we investigated the effects of the inhibitor ISRIB, ISRIB being an activator of the eIF2 guanine nucleotide exchange factor [38]; the results showed that ISRIB treatment inhibited upregulation of the protein ATF4 in cells treated that had been with 24S-OHC or thapsigargin (Fig. 6a). The 24S-OHC-induced decrease in puromycin-labeled protein levels was significantly suppressed by ISRIB, without affecting calreticulin levels. We also observed that ISRIB suppressed SG formation induced by 24S-OHC or thapsigargin (Fig. 6b, c) without affecting TIA1 and G3BP1 expression (Fig. 6a). Moreover, 24S-OHC-induced cell death could be inhibited to greater degree by ISRIB than by GSK2606414 (Fig. 6d). These results indicated that activation of the ISR accompanied by repression of protein synthesis and formation of SG was implicated in 24S-OHC-induced cell death.

## Discussion

ISR is influential in maintaining or restoring proteostasis in response to various physiological and pathological conditions [2, 6]. ISR signaling is evoked by various stress inputs, such as ER stress, amino acid deprivation, viral infection, and oxidative stress. ISR signaling reduces global protein translation, and induces expression of specific mRNAs to assist in cell survival and recovery from stress [2]. When the stress is severe in intensity or duration, the ISR triggers cell death to eliminate the damaged cell [39]. The present study demonstrated that cholesterol metabolite 24S-OHC activated the ISR signaling pathway, including the PERK and GCN2 branches. Both PERK and GCN2 activation, as well as eIF2α activation downstream therefrom, were suppressed by an ACAT inhibitor, suggesting that it is the ACAT1-catalyzed esterification of 24S-OHC that is responsible for the activation of ISR. We further demonstrated that inhibition of the ISR signaling pathway significantly suppressed cell death, suggesting that ISR has an essential role in 24S-OHC-inducible cell death.

We previously reported that ER dysfunction caused by accumulation of 24S-OHC esters and the accompanying decrease in ER chaperones induces PERK–UPR activation [32]. Activation of GCN2 occurs due to presence of uncharged tRNAs that may accumulate as a result of the depletion of their cognate amino acids or in response to other stressors such as oxidative stress and UV-B irradiation [40, 41]. Furthermore, GCN2 is also activated by stalling and collisions of elongating ribosomes during certain stresses [42]. It has been reported that 25-OHC-mediated GCN2 activation is independent of LXR and SREBPs but may involve oxidative stress and/or depletion of cysteine [37]. Because we previously showed that oxidative stress did not increase in 24S-OHC-treated cells and that 24S-OHC-inducible cell death was not suppressed by cysteine supplementation by N-acetylcysteine [25], neither oxidative stress nor cysteine appears to be involved in 24S-OHC-induced GCN2 activation. As it is possible that deprivation of other amino acid(s) or ribosome stalling is implicated in 24S-OHC-induced GCN2 activation, additional studies are warranted to determine the exact stresses to which GCN2 responds. Because the effects of GCN2iB on 24S-OHC-induced SG assembly, translational attenuation, and cell death were not evident, it was thought that the PERK branch plays a central role in the ISR signaling. We do not exclude the possibility that not only PERK and GCN2 but also other eIF2α kinases, e.g., HRI and/or PKR, may be involved in the 24S-OHC-inducible ISR.

As we also observed that the global blockade of protein synthesis was significantly restored by ISRIB, indicating that a part of this translational downregulation is likely to be a downstream consequence of eIF2α phosphorylation. We postulate that, because an inhibitor of PERK, GCN2, and ISR cannot suppress ER membrane disruption, the inhibitory effects of GSK2606414, GCN2iB, and ISRIB on translational attenuation and cell death were partial. As we reported previously, RIDD might partially account for the observed decrease in newly synthesized proteins [32]. Furthermore, as it has been indicated that cellular stress can cause the downregulation of global protein translation independently of eIF2α phosphorylation [43], other mechanisms might be involved in the 24S-OHC-induced dramatic decrease in nascent proteins. We do not exclude the possibility that 24S-OHC downregulated the mechanistic target of rapamycin (mTOR) signaling pathway, which promotes protein synthesis by phosphorylating 4E-BPs and p70 S6 kinase 1 [44]. It is also possible that the downregulation of protein synthesis was caused by the 24S-OHC-induced decrease in the rough ER.

As SG formation is caused by eIF2α phosphorylation, various stressors that activate the ISR signaling pathway are involved in the triggering of SG formation [8–11]. This study found that the cholesterol metabolite 24S-OHC also induced SG formation via the ISR pathway. LLPS of mRNP complexes occurring due to reversible, low-affinity interactions plays an essential role in the formation of such membraneless SGs. With the resolution of stress, the disassembly of SGs leads to the resumption of protein synthesis. We showed that the 24S-OHC-induced decrease in puromycin-labeled protein levels could be mitigated through inhibition of ISR, suggesting that SG formation might be involved in the 24S-OHC-induced repression of *de novo* protein synthesis. Defects in SG dynamics have been linked to various degenerative disorders, such as ALS and AD [10, 45]. We found that 24S-OHC caused ALS-associated TDP-43 to be incorporated into SGs. Because SGs have been implicated in Tau aggregation [45, 46], it would be interesting to investigate whether 24S-OHC, which is increased in patients with AD [20–22], induces recruitment by SG of Tau. It is of note that, independent of translation reprogramming, SG formation negatively regulates the apoptotic response. For example, sequestration of the receptor of activated protein C kinase 1 (RACK1) in SGs limits activation of the p38 and JNK MAPK apoptosis-triggering pathways, thereby preventing apoptosis [47]. It is also known that recruitment of mTOR complex 1 (mTORC1) component raptor to SGs is able to prevent mTORC1-hyperactivation-induced apoptosis [48]. It would therefore be of interest to investigate whether 24S-OHC-induced SG formation suppresses activation of apoptosis signaling and instead results in induction of caspase-independent unconventional cell death.

**Fig. 7:**
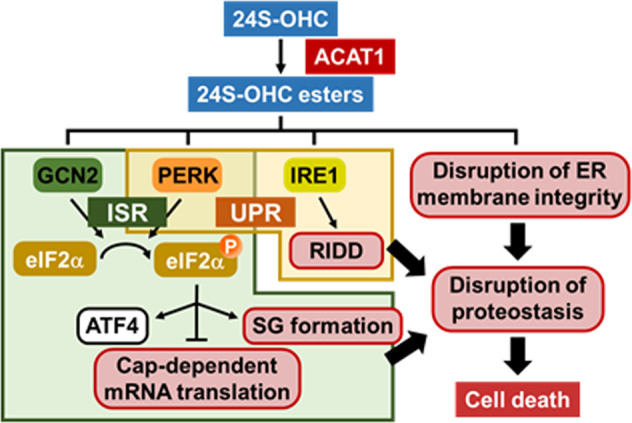
Schematic representation of mechanism proposed for 24S-OHC-induced cell death in SH-SY5Y cells. A detailed explanation of this model is provided in the Discussion.

Regulated cell death (RCD) is a controlled cellular process in which a cell activates its own molecular autodestruction machinery [49]. RCD is essential to the proper development and maintenance of tissue homeostasis; deregulation of RCD has been implicated in the pathogenesis of a number of diseases. Accumulating experimental evidence has revealed that there are multiple types of cell death [49]. We previously demonstrated that SH-SY5Y cells treated with 24S-OHC exhibited features that were neither apoptotic nor necrotic [25]. Based on the current study and previous findings, we propose the existence of a machinery for 24S-OHC-induced cell death in SH-SY5Y cells which is as depicted in Fig. 7. Namely, ACAT1-catalyzed 24S-OHC esterification occurring in the ER that serves as initial key pro-cell death event to evoke: (i) disruption of ER membrane integrity; (ii) activation of pro-death UPR signaling (including activation of both the IRE1 branch and the PERK branch); and (iii) activation of pro-death ISR signaling by way of either PERK or GCN2 activation. Phosphorylation of eIF2α which is involved in ISR signaling suppressed 5′ Cap-dependent protein translation and induction of SG formation. Cooperation among ISR, UPR, and disruption of ER membrane integrity cause disruption of proteostasis and ultimately result in induction of cell death by 24S-OHC. Based on the foregoing distinguishing features, we concluded that 24S-OHC-induced cell death may be an unconventional type of RCD. There is a growing body of evidence which suggests that accumulation of oxysterols is linked with the pathophysiologies of, and may serve as potential biomarker for, various diseases [50, 51]. Further studies will clarify the significance of the various features that make 24S-OHC-induced cell death distinct from oxysterol-induced cell death more generally.

## Materials and methods

### Materials

24S-OHC [52] was dissolved in EtOH (Wako, Osaka, Japan). F12511 was the generous gift of Kowa (Aichi, Japan). Thapsigargin and CHX were purchased from Wako (Osaka, Japan). GSK2606414 and ISRIB were from Cayman Chemical (Ann Arbor, MI, USA). GCN2iB was from MedChemExpress (Monmouth Junction, NJ, USA). Thapsigargin, GSK2606414, CHX, and ISRIB were dissolved in dimethyl sulfoxide (DMSO; Wako). The following antibodies were from commercial sources: anti-PERK (Cat# 3192), anti-phospho-eIF2α (Cat# 3398), anti-eIF2α (Cat# 5324), and anti-GCN2 (Cat# 3302) were from Cell Signaling (Danvers, MA, USA); anti-β-actin (Cat# A5441) was from Sigma-Aldrich (St. Louis, MO, USA); anti-TIA1 (Cat# 12133-2-AP), anti-ATF4 (Cat# 10835-1-AP), and anti-TDP-43 (Cat# 12782-2-AP) were all from Proteintech (Chicago, IL, USA); anti-G3BP1 (Cat# 611126) was from BD Biosciences (Franklin Lakes, NJ, USA); anti-phospho-GCN2 (Cat# ab75836) was from Abcam (Cambridge, UK); and anti-puromycin (Cat# MABE343) was from Merck Millipore (Burlington, MA, USA); All other chemicals, of analytical grade, were obtained from Sigma-Aldrich or Wako.

### Cell treatment and determination of cell viability

Human neuroblastoma SH-SY5Y cell line was purchased from the European Collection of Cell Cultures (Salisbury, UK) and routinely maintained as described previously [32]. The cultured cells were treated with 30 μM or 50 μM 24S-OHC or with 1 μM thapsigargin for the indicated period. EtOH (0.5%) was used for vehicle control treatments. For ACAT inhibition, cells were pretreated with 5 μM F12511 for 15 min before further treatment. Cells were also treated with 100 μM CHX, 0.5 μM GSK2606414, 0.5–1 μM GCN2iB, or 200 nM ISRIB in the presence or absence of 50 μM 24S-OHC for the indicated periods. Cell Counting Kit-8 (Dojindo, Kumamoto, Japan) was used for determination of cell viability.

### Immunoblotting and measurement of protein synthesis using the SUnSET assay

Preparation of whole-cell extracts and immunoblotting were performed as described previously [32, 53]. For evaluation of *de novo* global protein synthesis, cells were exposed to 10 μg/ml puromycin for 15 min before lysis. Whole-cell lysates were immunoblotted with an antibody specific for puromycin to detect levels of puromycin-labeled proteins.

### Polysome profile analyses

Cells were treated with 100 μg/ml CHX for 10 min prior to being harvested. Cells washed with ice-cold PBS containing 100 μg/ml CHX were lysed in 550 μl of polysome lysis buffer (50 mM Tris-HCl at pH 7.5, 100 mM NaCl, 30 mM MgCl_2_, and 0.1% NP-40) containing a protease inhibitor cocktail, 100 μg/ml CHX and 40 U/ml RNase inhibitor (Takara Bio, Shiga, Japan) at 4 °C for 30 min. The lysate was centrifuged for 10 min at 13,000 ×g. The supernatant (500 μl) was loaded onto a 10–50% sucrose gradient in polysome lysis buffer. The gradient was subjected to centrifugation in a Beckman SW55Ti rotor at 100,000 xg at 4 °C for 1 h, following which fifty 100-μl fractions were collected from the top. Absorbance was measured at 260 nm for each fraction.

### Immunofluorescence staining

Immunofluorescence staining was performed as previously described [27]. Cover slips were incubated with anti-TIA1, anti-G3BP1, and anti-TDP-43 antibodies for 3 h, and then incubated with the respective Alexa Fluor-conjugated secondary antibodies for 1 h at room temperature. Confocal fluorescence images were acquired with a Zeiss LSM710 confocal laser microscope having an oil objective lens, and were further processed using LSM Software ZEN2009.

### Statistics and reproducibility

Unless otherwise indicated, data shown are the mean ± SD of at least three independent experiments. Statistical significance was calculated by analyzing variance using ANOVA and the Tukey–Kramer multiple comparisons test. A significance threshold of *P* < 0.05 was employed.

## Supplementary information

Supplemental figure

## Data Availability

Full blots are available in the attached Supplementary Information. All other data are available from the corresponding authors upon reasonable request.
