# Supplementary material for: Integrated stress response is involved in the 24(S)-hydroxycholesterol-induced unconventional cell death mechanism
Source: Cell Death Discov. 2022 Oct 4;8:406. doi: 10.1038/s41420-022-01197-w (PMC9532424; doi:10.1038/s41420-022-01197-w)

Figure S1

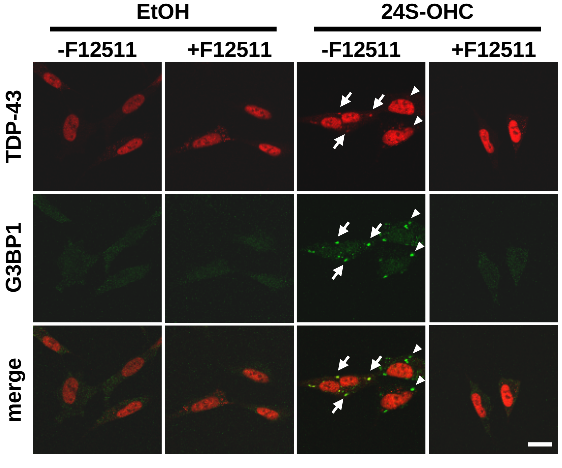

Figure S1. 24S-OHC induced TDP-43 recruitment to SGs in SH-SY5Y cells. Cells grown on cover slips were pretreated with 5 μM F12511 for 15 min and then exposed to 50 μM 24S-OHC for 3 h. Cells were processed for immunofluorescence staining with an antibody against TDP-43 or G3BP1. Representative confocal images are shown. The white arrows indicate colocalization. The white arrowheads indicate G3BP1 only. Scale bar: 20 μm.

Figure S2 (unprocessed blots)

Figure 1a

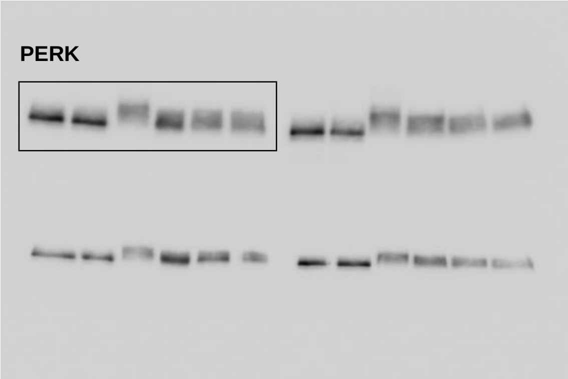

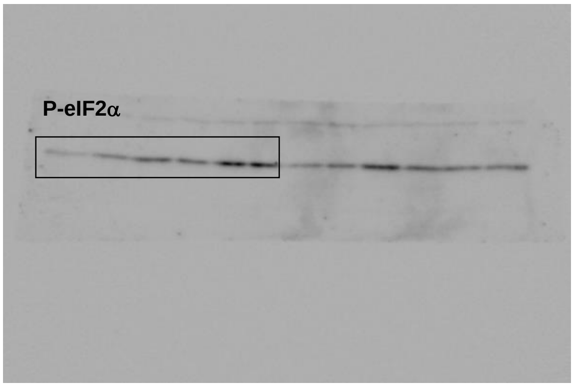

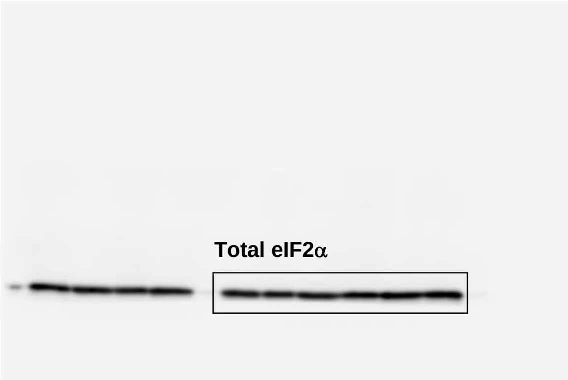

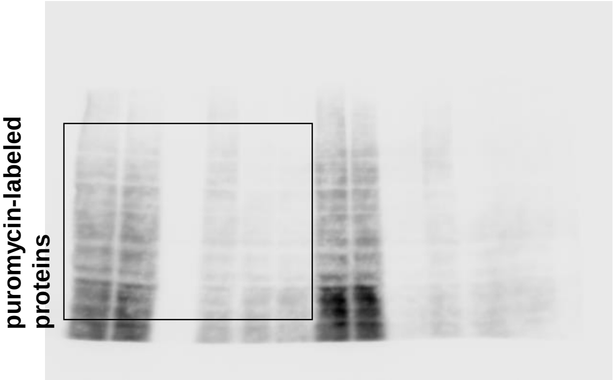

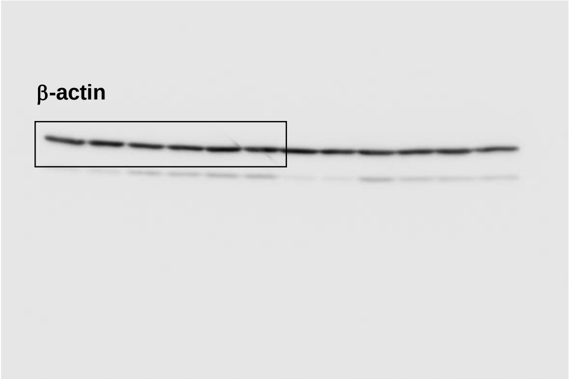

Figure S2 (unprocessed blots)

Figure 2c

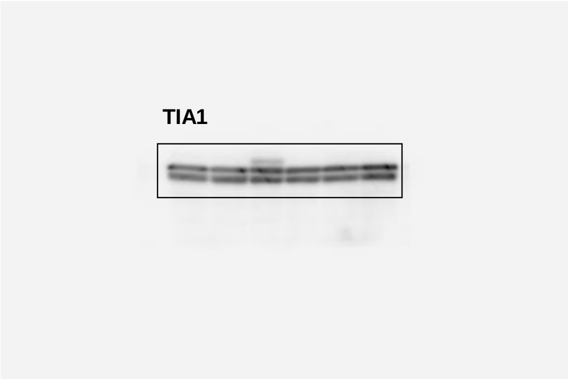

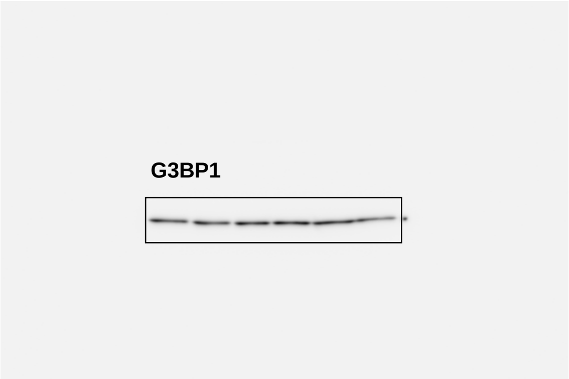

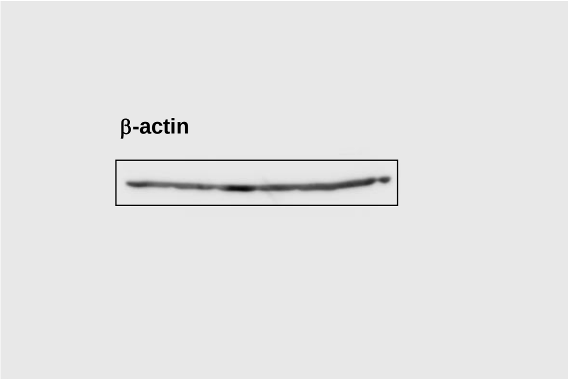

Figure S2 (unprocessed blots)

Figure 3c

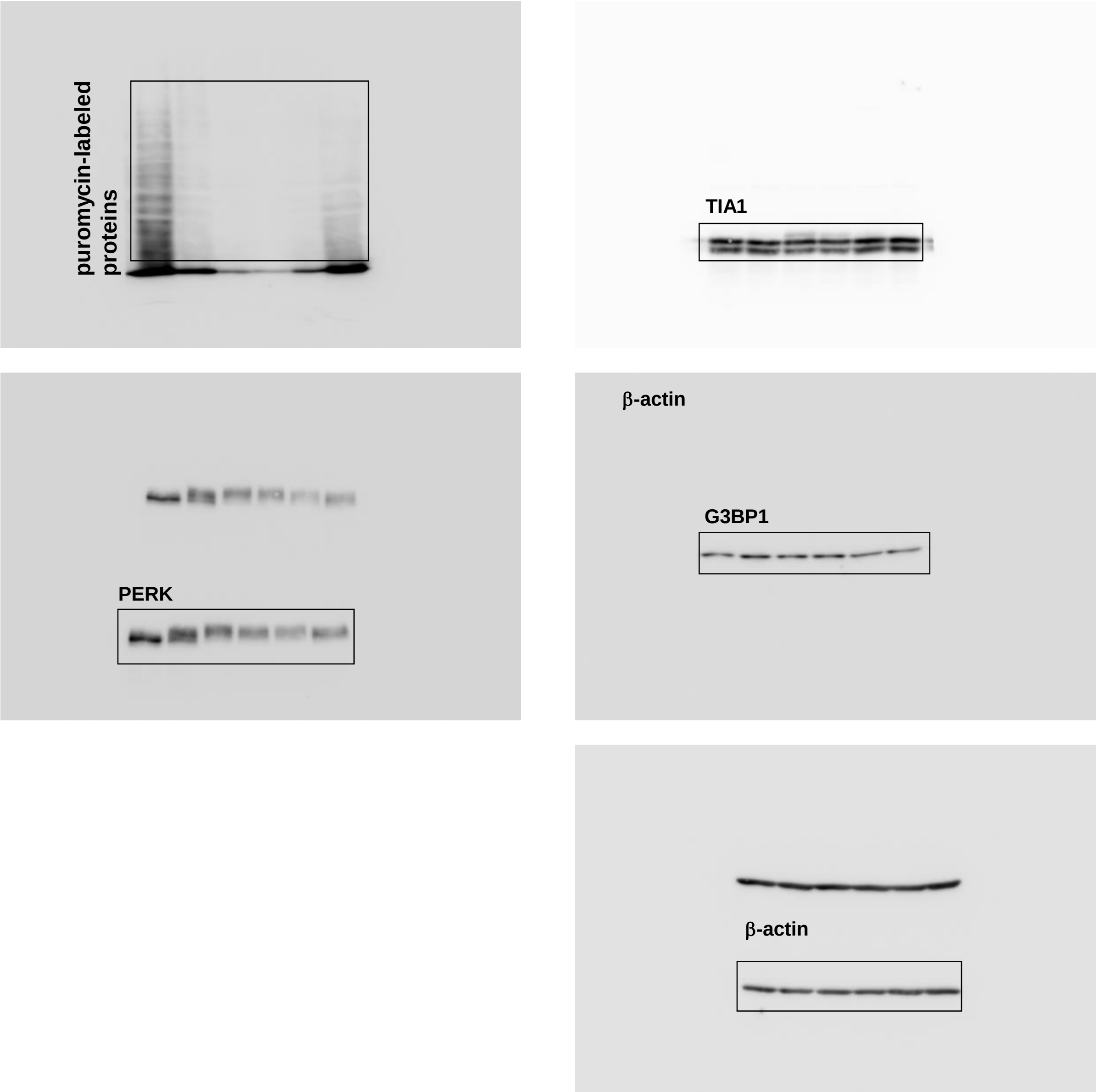

Figure S2 (unprocessed blots)

Figure 4a

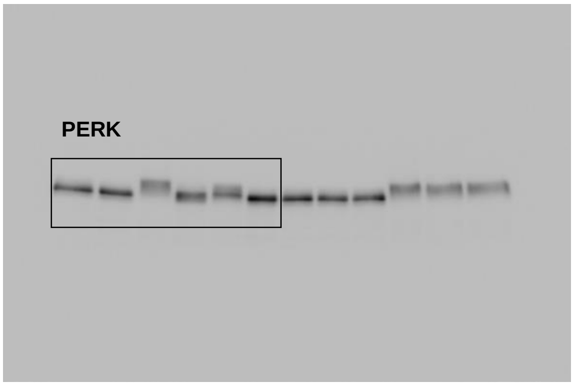

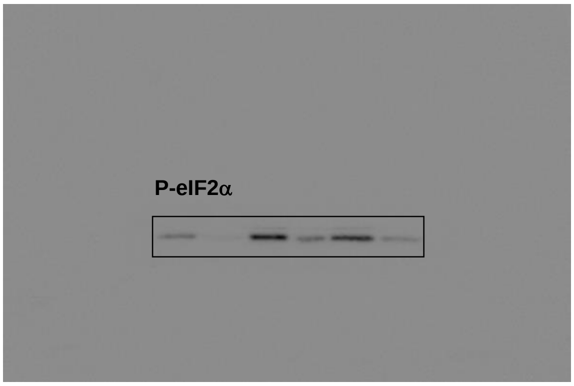

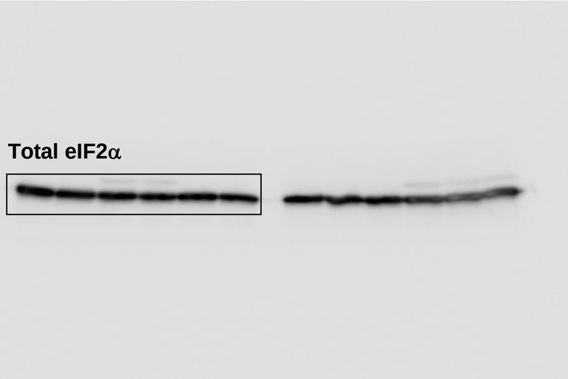

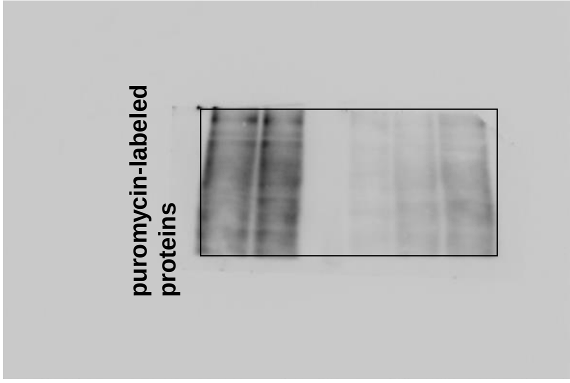

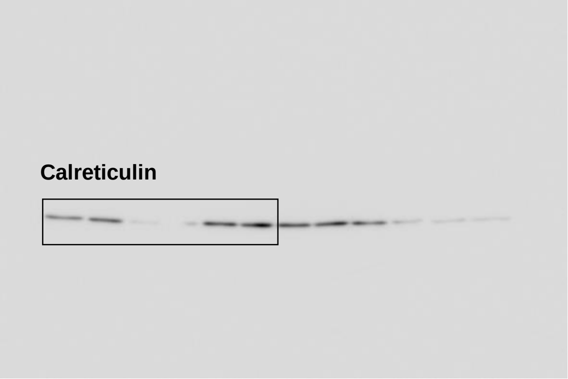

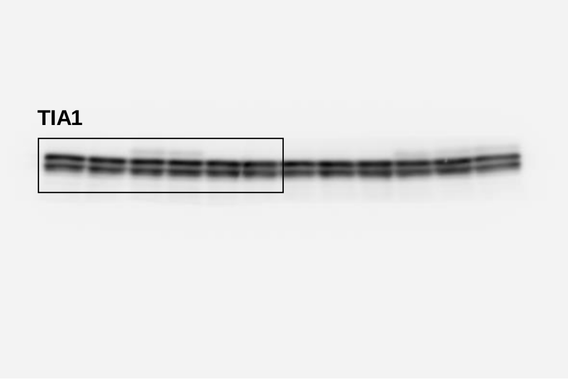

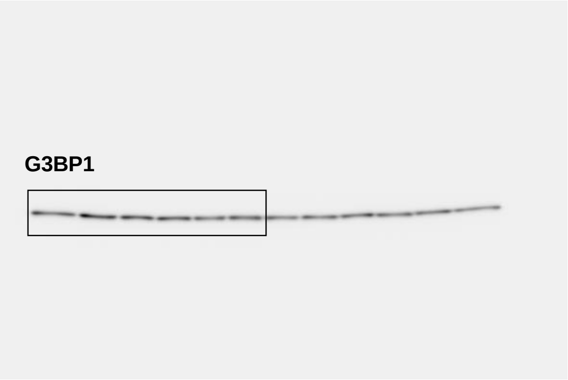

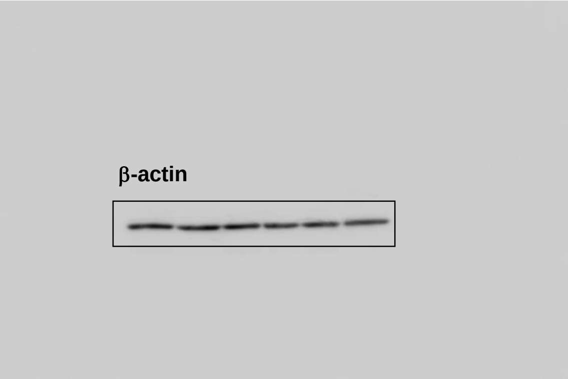

Figure S2 (unprocessed blots)

Figure 5a

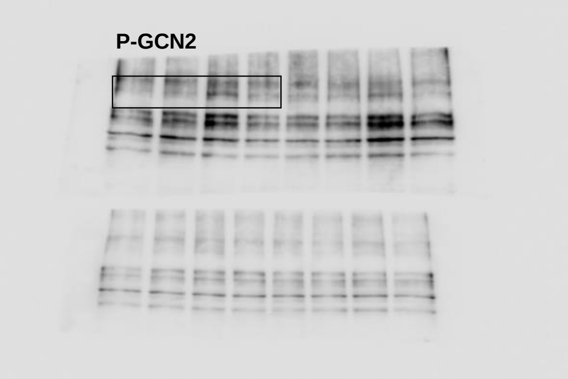

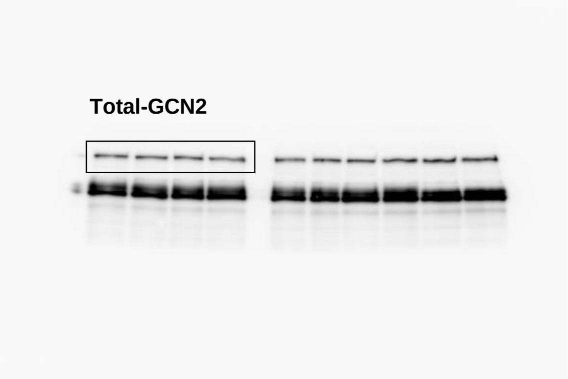

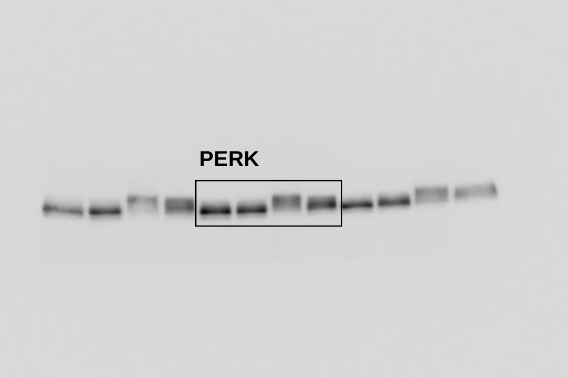

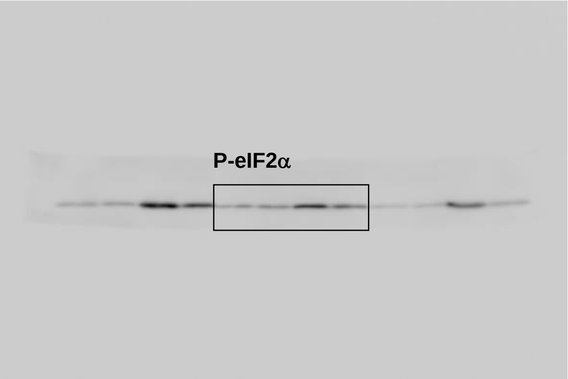

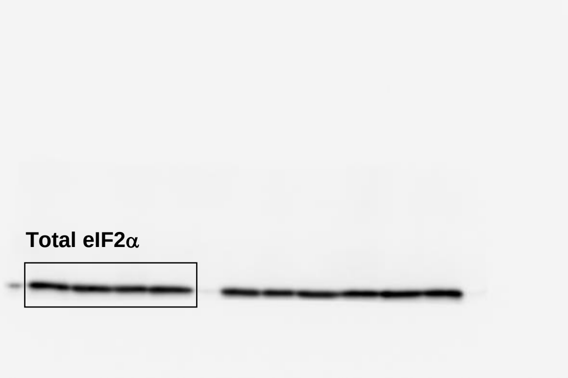

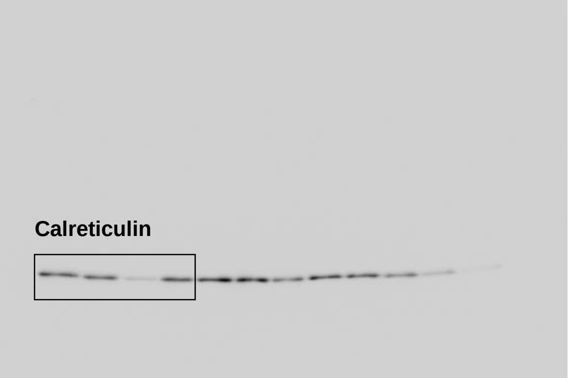

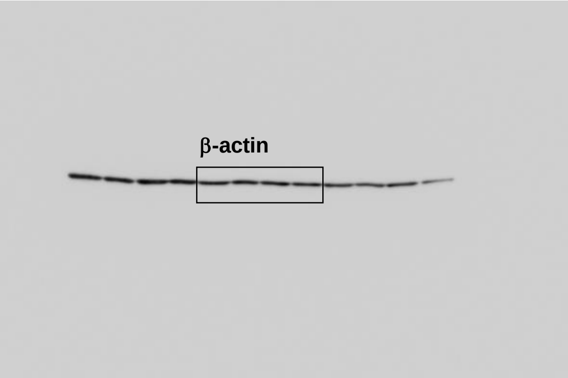

Figure S2 (unprocessed blots)

Figure 5b

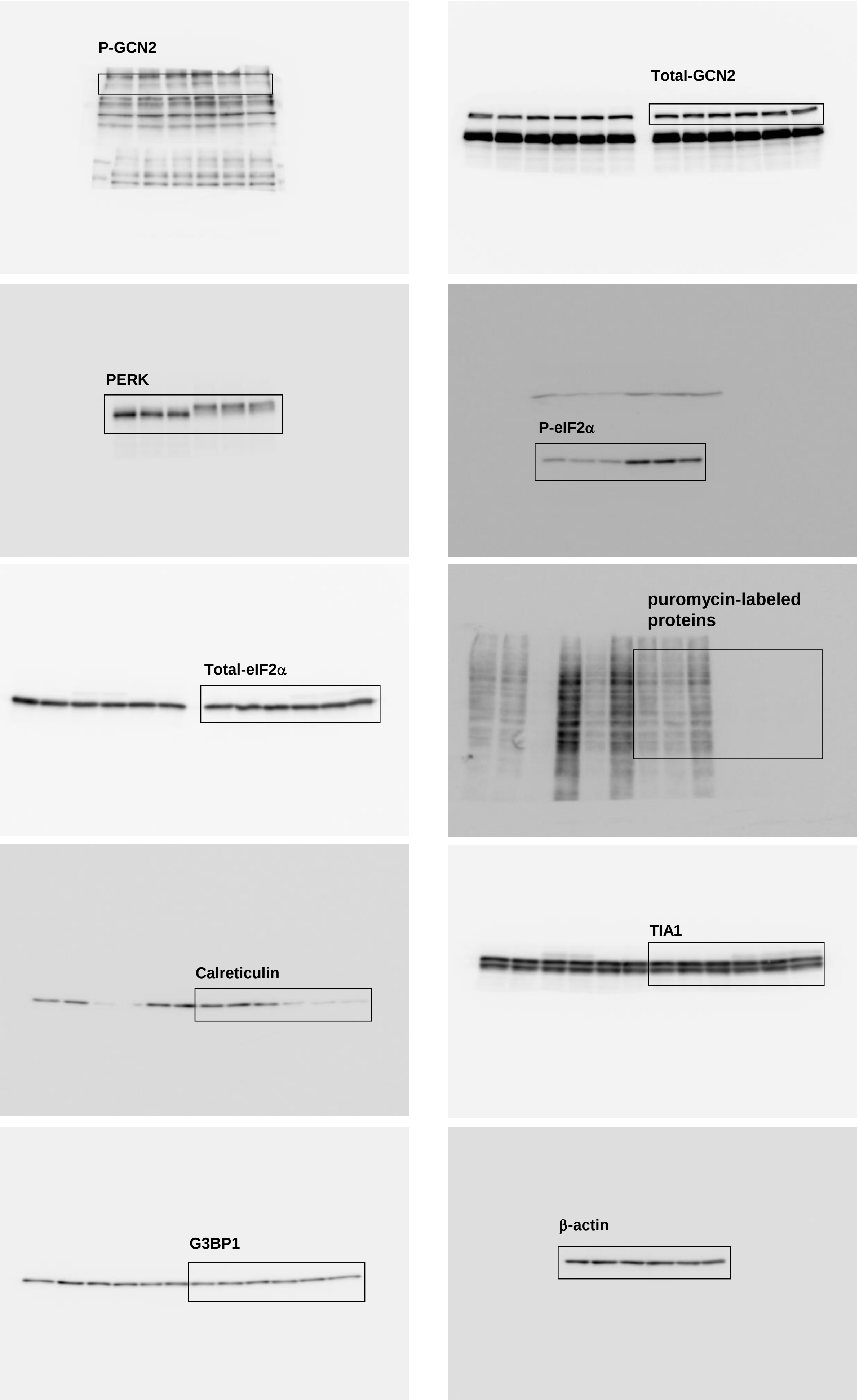

Figure S2 (unprocessed blots)

Figure 6a

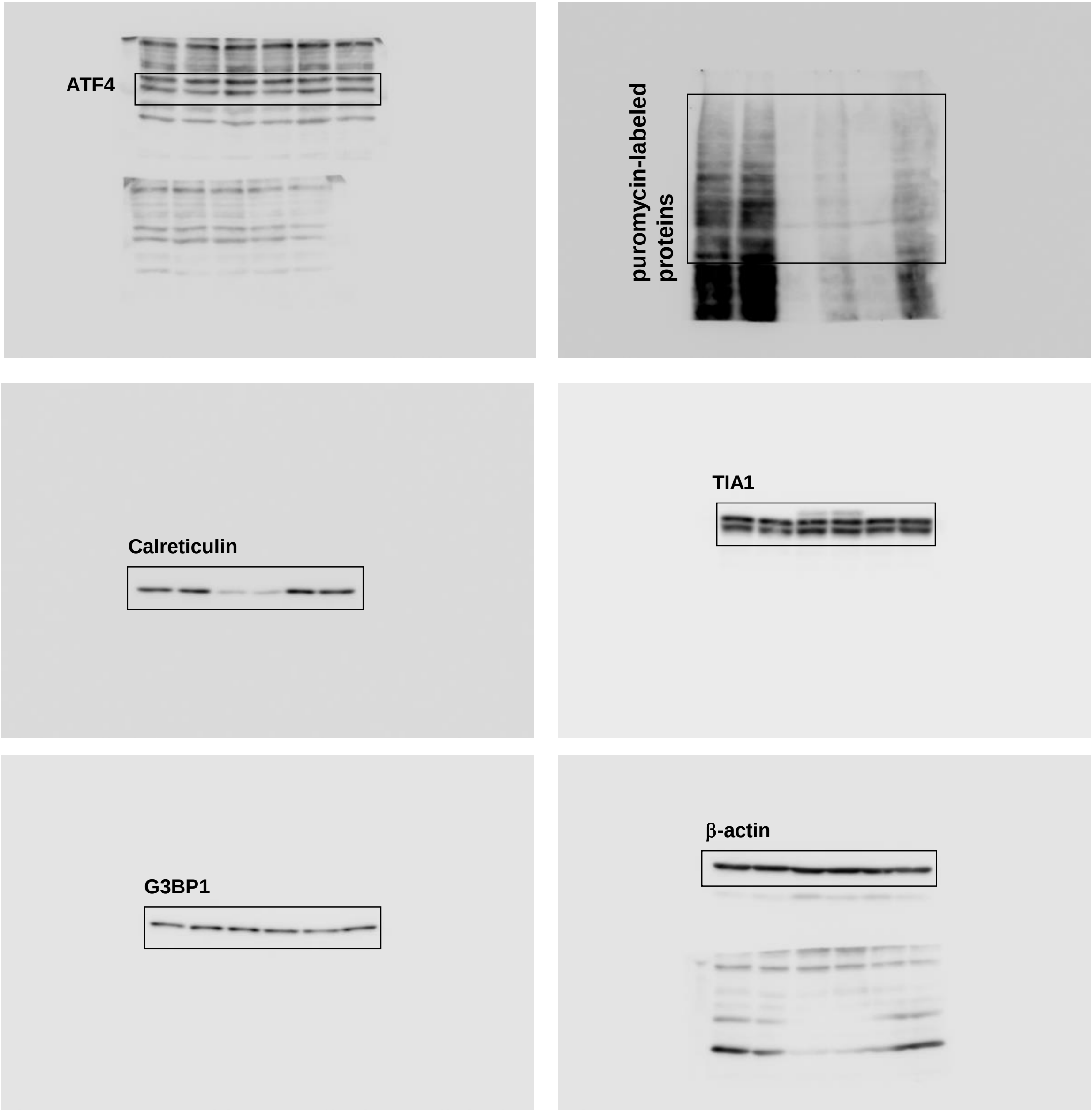

Supplement: Supplementary file 1 — Supplemental figure [file 41420_2022_1197_MOESM1_ESM.pdf]
